# Does clicker training reduce stress in shelter cats?

**DOI:** 10.18849/ve.v7i4.484

**Published:** 2022-11-18

**Authors:** Saskia Travers+

**Keywords:** BEHAVIOUR, CLICKER TRAINING, ENRICHMENT, FELINE, SHELTER, STRESS

## Abstract

PICO question

In domestic cats in a shelter setting, does clicker training decrease proxy measures of a stressed emotional state (behavioural or physiological) compared to no clicker training?

Clinical bottom line

Category of research question

Treatment

The number and type of study designs reviewed

Two papers were critically reviewed. One was a prospective quasi-randomised clinical trial, the other was a quasi-experimental before-and-after study with each cat being its own control

Strength of evidence

Weak

Outcomes reported

Both papers reported a decrease in behavioural stress indicators in shelter cats following a clicker training programme. Not all of these decreases were statistically significant and there are large issues with confounding factors in both papers

Conclusion

Preliminary evidence suggests that clicker training can be implemented as one form of enrichment to reduce stress in shelter cats alongside other means. Further evidence is required to demonstrate superiority to other forms of enrichment to reduce stress in shelter cats, especially given the practical limitations of implementing such a programme in most shelters

How to apply this evidence in practice

The application of evidence into practice should take into account multiple factors, not limited to: individual clinical expertise, patient’s circumstances and owners’ values, country, location or clinic where you work, the individual case in front of you, the availability of therapies and resources.

Knowledge Summaries are a resource to help reinforce or inform decision making. They do not override the responsibility or judgement of the practitioner to do what is best for the animal in their care.

## Clinical scenario

You are a shelter medicine veterinarian working in a busy cat adoption centre. You are in a staff meeting discussing novel means of enrichment for the cats. A colleague mentions that she has heard of other shelters clicker training cats to reduce stress and improve adoptability. Your initial thoughts are that clicker training would take a significant amount of time in such a busy shelter, but you are intrigued by the idea and the potential benefits. You decide to examine the evidence to assess whether clicker training can decrease stress in shelter cats, before you and your colleagues decide whether to implement it.

## The evidence

A focused search found two papers relevant to the PICO question, examining the use of clicker training in the shelter environment alongside measures of stress.

One was a prospective, randomised clinical trial (Gourkow & Phillips, 2016) and the other was a before-and-after study with each cat acting as its own control (Grant & Warrior, 2019).

## Summary of the evidence

**Gourkow & Phillips (2016):** 

Population:	Domestic cats in a shelter in Vancouver, Canada. All cats had either been surrendered by their owners or found as strays. All cats were aged 6 months or older. They were clinically healthy with no signs of upper respiratory disease or injury on recruitment for the study. This study was part of a larger study reported in three separate articles, assessing the effects of different interventions depending on the assessed mood of the cats using an ethogram (Gourkow et al., 2014a). This initially involved classifying 250 cats as ‘frustrated’, ‘anxious’ or ‘content’. The 15 cats deemed ‘frustrated’ were recruited for this study and underwent clicker training, assessing whether cognitive enrichment is beneficial for these cats. The other cats were deemed ‘anxious’ (139 cats) or ‘content’ (96 cats) and their interventions are described elsewhere (Gourkow et al., 2014b; and Gourkow & Phillips, 2015). Cats were deemed ‘frustrated’ if they demonstrated any of the following behaviours during more than 10% of their awake time: vocalisation, escape attempts, visual scanning, pushing objects, pacing, or short bouts of aggression during human interaction.
Sample size:	15 ‘frustrated’ cats.
Intervention details:	This study consisted of two treatment groups: Treatment (n = 7): Underwent training sessions four times a day for 10 days by one experimenter. The paper does not state whether they also received interaction outside of training sessions. Control (n = 8): These cats were completely ignored and did not receive any interaction at all, neither by shelter staff during routine care nor the experimenters. Cats were randomly allocated to each group by systematic sampling, through alternating which group an incoming 'frustrated’ cat would be allocated to (treatment or control) in order of admission to the shelter. In this way, every other cat was allocated to the ‘control’ group. The experiment took place in a separate unit to the rest of the shelter. Cats were housed in stainless steel cages (76 × 76 × 71 cm) within a separate housing unit to the rest of the shelter. For the Treatment group, training took place in a 4 m2 training room adjacent to where the cats were kept. Cat behaviour were assessed via daily video footage and classified as ‘content’, ‘frustrated’ or ‘apathetic’ by use of an ethogram, previously developed by the same authors (Gourkow et al., 2014a).Cats in the treatment group were first conditioned to anticipate a treat with a ‘click’ sound and then trained to perform ‘give me five’ through shaping (rewarding approximations of the behaviour until the desired behaviour was performed). This study examined two responses to stress: behavioural (mood rating using an ethogram) and physiological. The physiological data consisted of secretory IgA (s-IgA) assays on faeces, and whether cats developed upper respiratory disease (considered to be a proxy measure of stress-induced viral shedding). Every stool produced was collected, weighed and immediately stored at -40°C. IgA was measured using an enzyme-linked immunosorbent assay following homogenisation of faeces.
Study design:	Prospective, quasi-randomised clinical trial.
Outcome Studied:	Behavioural indicators of stress (subjective). The authors observed cats using focal sampling; 10 minutes per hour for the 10 days. Behaviours were categorized according to the ethogram if said behaviours were observed for more than 10% of the awake time. Number of cat days, and proportion within each group, rated as ‘frustrated’, ‘content’ or ‘apathetic’. Mean time taken to reach a ‘content’ state for each group. Whether cats in the treatment group are more likely to remain ‘content’ once attained, compared to the control group, using a Cox proportional hazards model (Cox, 1972). This is a type of survival analysis which in this context is being used to measure time before the mood rating changes. Physiological indicators of stress (objective): Daily faecal s-IgA levels. Incidence of upper respiratory disease, presumed to be a marker of stress-induced viral shedding.
Main Findings (relevant to PICO question):	Behavioural indicators of stress: Mood rating per cat day, and proportion within each group: The treatment group were significantly (P = 0.002) more likely to have cat days rated as ‘content’ than cats in the control group. Within the control group, 49/67 (73%) of cat days were rated as negative. Of these, 41/67 (61.2%) were deemed ‘frustrated’ and 8/67 (11.9%) were considered ‘apathetic’. In the treatment group, 28/55 (51%) of cat days were rated negatively. All were deemed ‘frustrated’. Time taken to reach a ‘content’ rating: On average, cats in the treatment group were rated ‘content’ slightly earlier than those in the control group (3.2 days (±1.3 days) vs 4.4 days (±0.3 days)). However, this finding was not statistically significant. Hazard ratio (HR): 3.64, confidence interval (CI): 0.85–15.67, P = 0.08 (not statistically significant). Whether treated cats are more likely to remain ‘content’ once attained compared to the control cats: Treated cats appeared to be 3.6 times more likely to remain ‘content’ once attained compared to the control group, however this result is not statistically significant. HR: 3.64, CI: 0.85–15.67, P = 0.08 (not statistically significant). Physiological indicators of stress: Daily stool s-IgA levels: Treated cats had significantly (P = 0.03) greater S-IgA levels than those in the control group. 6.73 ± 0.47 µg/g for the treated cats, 6.04 ± 0.68 log_e_ µg/g in the control group. Incidence of upper respiratory disease: Cats in the control group were significantly (HR: 2.37, CI: 1.35–4.15, P = <0.0001) more likely to develop upper respiratory disease than cats in the treatment group, as determined by the Cox Proportional Hazards Model (Cox, 1972).
Limitations:	This study had a control group, but the control group is not particularly meaningful because these cats had no interaction at all while the treatment group were let out of their small cages into a larger room and interacted with at least four times a day. The lack of enrichment in the control group creates a severe confounding effect when assessing whether clicker training itself is beneficial, or whether it is simply the interaction or even being outside of a cage. This is an important aspect that must be considered when interpreting the results; the authors are comparing clicker training to no enrichment at all. Small sample size (seven cats in the treatment group, eight in the control group). This means that individual differences, such as personality and food motivation, are more likely to influence the results. This study only examined cats deemed to be ‘frustrated’, limiting the applicability of the results. The paper does not state which experimenter assessed the cats’ emotional states based on the video footage, raising the possibility of bias if one experimenter did both the training and behavioural assessment.

**Grant & Warrior (2019):** 

Population:	Twelve domestic cats at the RSPCA’s Oxfordshire rehoming centre, UK. The ages of the cats ranged from 1–11 years. Ten of these cats were relinquished by their owners and two were former strays. The length of stay prior to the training programme ranged from 2–25 days.
Sample size:	12 cats.
Intervention details:	All singly housed cats residing in the cattery at the time of the study were subjected to the clicker training programme. Behaviour assessments were carried out before the clicker training programme, and after the six clicker training sessions had been completed. All cats underwent the same intervention with no control group. The study utilised a quasi-experimental (i.e. non-randomised, defined as per Harris et al., 2006), uncontrolled before-and-after study design. This means that there was no separate control group, as each cat acted as its own control. The term is used to contrast with controlled before-and-after studies, where there is a control group not receiving the intervention allowing for comparison (Goodacre, 2015). The experiment was carried out as follows: Behavioural observation – before the training programme: Each cat had a 10 minute period of observation prior to the training programme beginning. This consisted of: 10 minute video recording (via a smartphone) to allow observation of behaviour and subsequent categorisation. The experimenter recording did not interact with the cats during this time. Behaviour was classified as follows, based on an ethogram created by Stanton et al. (2015): Exploratory: investigate surroundings (such as sniffing objects or manipulating with paws). Play: interacting with objects in a playful context. Inactivity: stationary in a sitting, lying or standing position. Other: neutral behaviours not fitting into the above categories (normal grooming, drinking, eating, defecation). Recording the amount of time spent at the front and back of the cage. Human Approach Test (HAT), based on the work of Arhant & Troxler (2017). The experimenter presented their hand to the cat and recorded whether the cat made contact with, investigated or simply did not withdraw from their hand (contact possible [CP]). If the cat withdrew, showed signs of aggression towards or froze in reaction to their hand, this was recorded as no contact possible (NCP). Training programme: Consisted of 10 minute training sessions, three times a week for 2 weeks (six sessions total). The training was as follows: ‘Charging’ the clicker: clicking then presenting a food reward, until the cat began to anticipate food after the click. The distance was increased between the trainer and the cat, so the cat had to travel towards the trainer to obtain the food reward. The trainer added a vocal cue by calling the cat’s name, marking the behaviour when the cat was close to the trainer. For fearful cats, food was tossed near the cat and the clicker marked the cat approaching the food, gradually building up on this until the cat approached the trainer. Behavioural observation after the training programme: This was done 2 days after finishing the programme and was done by a person unfamiliar to the cats, in the same way as before training: 10 minute video recording (via a smartphone) to allow observation of behaviour and subsequent categorisation as ‘exploratory’, ‘play’, ‘inactivity’ or ‘other’. Recording time spent at the front and back of the cage. HAT, recording whether contact was possible or not. Each pen had an indoor area and an outdoor area, which the cat accessed via a small opening. The indoor area was not visible to visitors and therefore one of the primary aims of the study was to encourage cats to spend more time in the outdoor area. In addition to clicker training, cats continued to have socialisation sessions with volunteers multiple evenings a week. The cats in the study remained available for rehoming throughout, and as a result several cats were excluded due to rehoming before the clicker training programme had finished (the authors do not state how many). A paired t-test was used for recording the time spent in behavioural categories, and time spent at the front of the cage. A McNemar’s test with a 2 x 2 contingency table was used for the results of the HAT. This test is used in before-and-after studies to determine whether the proportions in two samples from the same individual are equal (Morrison, 2010).
Study design:	Quasi-experimental, uncontrolled before-and-after study.
Outcome Studied:	All outcomes were objective: Time spent in the ‘exploratory’ behavioural category, before and after the training programme (subjective, paired T-test). Time spent in the ‘inactive’ behavioural category, before and after the training programme. Time spent at the front of the cage, before and after the training programme. Whether contact was possible following the HAT, before and after the training test.
Main Findings (relevant to PICO question):	Results are not displayed numerically in this paper, but rather through bar charts. Therefore, all mean values have been rounded to the nearest integer on the charts (denoted with ~) to avoid misreporting of results. The bar charts have error bars but it is unclear whether these refer to the confidence interval or standard deviation, so the variability measures of the means are not reported here. There was a significant (t = 4.33; P = 0.001) increase in mean time spent in the ‘exploratory’ behaviour category after clicker training: Before the training programme, the mean time spent in exploratory behaviour was ~2 minutes. After the training programme, the mean time spent in exploratory behaviour was ~5 minutes. There was a significant (t = 4.33; P = 0.001) decrease in mean time spent in the ‘inactive’ behaviour category after the training programme: Before the training programme, the mean time spent in inactive behaviour was ~7 minutes. After the training programme, the mean time spent in inactive behaviour was ~1 minute. There was a significant (t = -4.67; P = 0.001) increase in the mean time spent at the front of the cage after clicker training: Before the training programme, the man time spent at the front of the cage was ~3 minutes. After the training programme, the mean time spent at the front of the cage was ~8 minutes. While more cats were classified as CP after the clicker training programme, the McNemar’s test did not find this statistically significant (P = 0.125). Before the training programme, 6/12 (50%) of cats had CP with the HAT. After the training programme, 10/12 (83.3%) had CP with the HAT.
Limitations:	Small sample size, meaning that individual differences between cats would have had a greater effect on the results. Several cats were excluded from the study because they were adopted before the intervention could be complete. This is understandable from an ethical standpoint but it also lessens the applicability of the intervention. There was no separate control group in this study, as each cat was used as its own control. It is therefore more difficult to ascertain whether the clicker training was responsible for the cats’ improved exploratory behaviours. Indeed, in human medicine uncontrolled before-and-after studies have been shown to overestimate the benefits of novel treatments (Sacks et al., 1982, cited in Goodacre, 2015) and at least one medical journal has limited publication of these studies to exceptional circumstances for this reason (Goodacre, 2015). It is worth considering that half of the cats (6/12) were CP in the HAT before the training intervention even began. There were several confounding factors, such as the cats continuing to receive interaction with volunteers during the study and the possibility that the cats became more accustomed to their environment over the 2 weeks. The authors acknowledge these limitations, but still interpret the improvement in four cats being CP post-training as being due to clicker training. The video recording post-training involved someone entering the cage to record the video on their smartphone, so it is possible that the cats spent more time on the outside of the pen because they were anticipating training and food. The authors attempted to reduce this effect by having an unfamiliar person record post-training. It is unclear whether the observed increase in exploratory behaviour was seen generally, when alone in the cage. Short observation period for behavioural assessment: 20 minutes total over the course of 2 weeks (one before training, one after). Does not state which experimenter assessed the cats’ behaviour based on the video footage, raising the possibility of bias.

## Appraisal, application and reflection

Cats were successfully clicker trained in both studies (Gourkow & Phillips, 2016; and Grant & Warrior, 2019), and at least one other paper has demonstrated that cats can be clicker trained in the shelter environment irrespective of other factors such as age (Kogan et al., 2017). Currently, the evidence suggests that cats can be clicker trained in the shelter environment but the evidence as to whether clicker training decreases stress in the shelter environment compared to other forms of cognitive enrichment is less clear, mainly due to the confounding factors at play in both papers.

The main limitations of both studies were the small sample sizes, lack of meaningful control groups and confounding factors. For instance, in the study by Grant & Warrior (2019), cats received socialisation by staff and volunteers in addition to training, and it is unclear whether the perceived increase in exploratory behaviour post-training was due to the cats anticipating training (and subsequently, food) when a person entered the cage to film the cats on a smartphone. Gourkow & Phillips (2016) took the approach of having a control group of cats that received no enrichment at all; this calls into question whether the perceived improvements were due to the clicker training or whether any form of enrichment (including the simple act of letting the cats out of the cage for training) would have created similar improvements, given that the control group cats without enrichment are going to be in a compromised emotional state regardless.

Clicker training animals is a time-consuming venture that many shelters would find difficult to do for every cat. Grant & Warrior (2019) attempted to make their training method as accessible as possible for that specific shelter environment, namely 10 minutes of training three times per week and aiming for the cats to spend more time in the outside area so visitors could see the cats. The authors speculated that encouraging these behaviours would improve adoptability, a view shared by Bollen (2015). Grant & Warrior (2019) did not have a separate control group and so it is difficult to assess whether clicker training had this desired effect, although research suggests that active and playful cats tend to be viewed more positively by potential adopters (Gourkow, 2001; Fantuzzi et al., 2010; Sinn, 2016; and Caeiro et al., 2017). An interesting subject for further research would be to assess whether clicker training cats results in a reduced length of stay in the shelter, although future studies of clicker training in cats could compare it to other means of cognitive enrichment to reduce the confounding factors. The external validity of these findings is limited by the fact that most cat rescue organisations have various resource constraints, and clicker training is a very labour-intensive means of enrichment for shelter cats.

Because of how labour-intensive a clicker training intervention is, it appears to be more appropriate as a means of enrichment for specific situations rather than a universal measure. Grant & Warrior (2019) tailored the clicker training programme for the specific cattery design (that is, encouraging cats to spend more time in the outside area), while Gourkow & Phillips (2016) only recruited cats they deemed ‘frustrated’ for the clicker training intervention. Shelter staff should take a tailored approach to feline enrichment as clicker training may not be suitable for every cat. For example, it has been suggested that a fearful cat may find increased human interaction stressful while a frustrated cat would be likely to benefit from it (Ellis, 2009). Clicker training can be employed alongside a variety of enrichment measures to reduce stress in shelter cats, many of which are less labour-intensive (for instance, puzzle feeders) and allow for cats to express their innate behavioural needs (such as providing scratching posts and play that mimics hunting).

To summarise, it is possible to clicker train cats in the shelter environment and there is weak evidence that it may reduce some proxy measures of stress, although confounding factors limit full attribution to clicker training. Clicker training can be used as a form of cognitive enrichment alongside other means, depending on the cats personality and presenting problems. However, unless further studies prove that clicker training is a superior form of enrichment to existing forms of enrichment (for example puzzle feeders, or having volunteers play with cats), and given the time requirements that may limit applicability of the studies, it seems more appropriate to suggest clicker training for certain situations and individual cats as part of a multidisciplinary approach, rather than as a universal recommendation for all cats in shelters.

## Methodology Section

**Search Strategy:** 

Databases searched and dates covered:	CAB Abstracts, via CAB Direct (1973–Week 4 2022)PubMed, via NCBI interface (1966–Week 4 2022)
Search strategy:	CAB Abstracts: cat OR cats OR feline OR “Felis catus” OR “Felis silvestris catus” OR felid* AND "clicker train" OR train* OR "clicker trained" OR "clicker training" OR “conditioned reinforc*” OR “secondary reinforc*” OR “successive approximation” OR shaping OR clickerANDshelter OR pound OR “rescue cent*” OR cattery AND stress OR stress* OR behavio* OR anxi* PubMed: ((cat OR cats OR feline OR "Felis catus" OR "Felis silvestris catus" OR felid*) AND (("clicker train" OR train* OR "clicker trained" OR "clicker training" OR “conditioned reinforc*” OR “secondary reinforc*” OR “successive approximation” OR shaping OR clicker)) AND (shelter OR pound OR "rescue cent*" OR cattery)) AND (stress OR stress* OR anxi* OR behavio*)
Dates searches performed:	30 Jan 2022

**Exclusion / Inclusion Criteria:** 

Exclusion:	Paper not in the English language. Abstract only papers, conference proceedings, policy, letters. Papers not relevant to the PICO.
Inclusion:	Peer reviewed research using a comparator of behaviour before and after a period of clicker training.

**Search Outcome:** 

Database	Number of results	Excluded – Does not answer PICO question	Excluded – Paper in a language other than English	Excluded – Abstract-only papers, conference proceedings, policy and letters	Excluded – Duplicates	Total relevant papers
CAB Abstracts	31	29	0	0	1	1
PubMed	17	16	0	0	0	1
Total relevant papers						2

## ORCID

Saskia Travers: https://orcid.org/0000-0002-8541-969X

## Conflict of Interest

The author declares no conflicts of interest.
